# No association between psychiatric symptoms and doses of anabolic steroids in a cohort of male and female bodybuilders

**DOI:** 10.1002/dta.3230

**Published:** 2022-02-21

**Authors:** Julio X. Amaral, Andrea C. Deslandes, Monica C. Padilha, Leonardo Vieira Neto, Luiz E. Osorio, Francisco Radler Aquino Neto, Marcelo S. Cruz

**Affiliations:** ^1^ Institute of Psychiatry, Psychology and Neurosciences (IoPPN) King's College London London UK; ^2^ Institute of Psychiatry Federal University of Rio de Janeiro (UFRJ) Rio de Janeiro Brazil; ^3^ Chemistry Institute‐Brazilian Laboratory of Doping Control (LBCD–LADETEC, IQ‐UFRJ) Federal University of Rio de Janeiro Rio de Janeiro Brazil; ^4^ Department of Internal Medicine Federal University of Rio de Janeiro (UFRJ) Rio de Janeiro Brazil

**Keywords:** aggressiveness, anabolic steroids, androgens, anxiety, mass spectrometry

## Abstract

The use of androgenic‐anabolic steroids (AAS) can be associated with psychiatric symptoms such as insomnia, anxiety and increased aggressiveness. Although dose‐dependent effects have been observed in some controlled studies, this association is not always seen in the ecological use of AAS. This study utilized WADA's steroid profile of suspicious use of AAS, urinary detection of AAS metabolites and measurement of sexual hormones to confirm recent use of AAS in a cohort of 103 bodybuilders (75 males, 28 females). The majority of participants (61.2%) presented symptoms of agitation, insomnia, increased aggressiveness or depression in the last 3 months. About one‐third of participants presented scores on the HAM‐A anxiety scale equivalent to moderate to severe symptoms of anxiety. A minority of participants (12.6%) presented high to moderate scores on the BPQ aggressiveness scale. The majority of participants (73.8%) presented hyperthymic temperament in the BRIEF‐TEMPS scale. There was no significant difference in the presence of psychiatric symptoms between males and females and no association between psychiatric symptoms and estimated weekly doses of AAS. A negative association was observed between scores on the BPQ scale (verbal aggression, anger and total score) and the time of AAS use. We discuss differences of AAS use between male and female bodybuilders and the screening of AAS use in the general population. Our findings highlight the importance of mental health awareness among people using AAS.

## Graphical abstract

This study describes patterns of anabolic steroid use and psychiatric symptoms in a cohort of male and female bodybuilders. Suspicious use of anabolic steroids was confirmed by screening analyses of urinary androgens and sexual hormones.

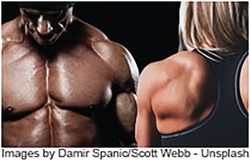

## 1 INTRODUCTION

Investigations of the psychiatric effects of androgenic‐anabolic steroids (AAS) have been conducted since the use of exogenous testosterone in the treatment of depression and melancholia was observed in the 1940s.^1^ Current evidence corroborates that the use of AAS can have beneficial effects on the central nervous system (CNS) such as reduction of depressive symptoms in men,^2^, ^3^ as well as detrimental psychiatric outcomes such as increased aggressiveness and anxiety.^4^, ^5^, ^6^, ^7^, ^8^, ^9^ Possible mechanisms that explain central effects of AAS include binding to and modulating the expression of CNS androgenic receptors,^10^, ^11^ and interfering with biosynthesis of endogenous neurosteroids.^12^ Although some controlled studies described a dose‐dependent association between AAS use and the onset of psychiatric symptoms,^13^, ^14^, ^15^ this association is not always observed in ecological use of AAS.^8^ In an attempt to reduce self‐report bias, recent studies have employed urinary analysis to detect and quantify the use of AAS in ecologic settings.^16^, ^17^ Since urinary analysis of androgens are not usually available in the clinical setting, seric sexual hormones affected by the use of AAS can also be used to investigate the likelihood of AAS use.^18^ Different patterns of AAS use can also impact ecological observations and comparisons between groups of AAS users. Bodybuilding athletes who use AAS are frequently reported among those using higher doses of AAS,^19^, ^20^, ^21^, ^22^ apart from having similar and comparable training routines and stressing factors such as bodybuilding competitions.^23^ The goal of this study is to investigate the use of AAS and the presence of psychiatric symptoms in a cohort of 102 Brazilian bodybuilders whose self‐reported use of AAS was confirmed by analysis of urinary androgens and seric hormones. We also compare the use of AAS and psychiatric symptoms between male and female participants and between those informing the use of different weekly doses of AAS.

## 2 MATERIALS AND METHODS

### 2.1 Study design

**FIGURE 1:**
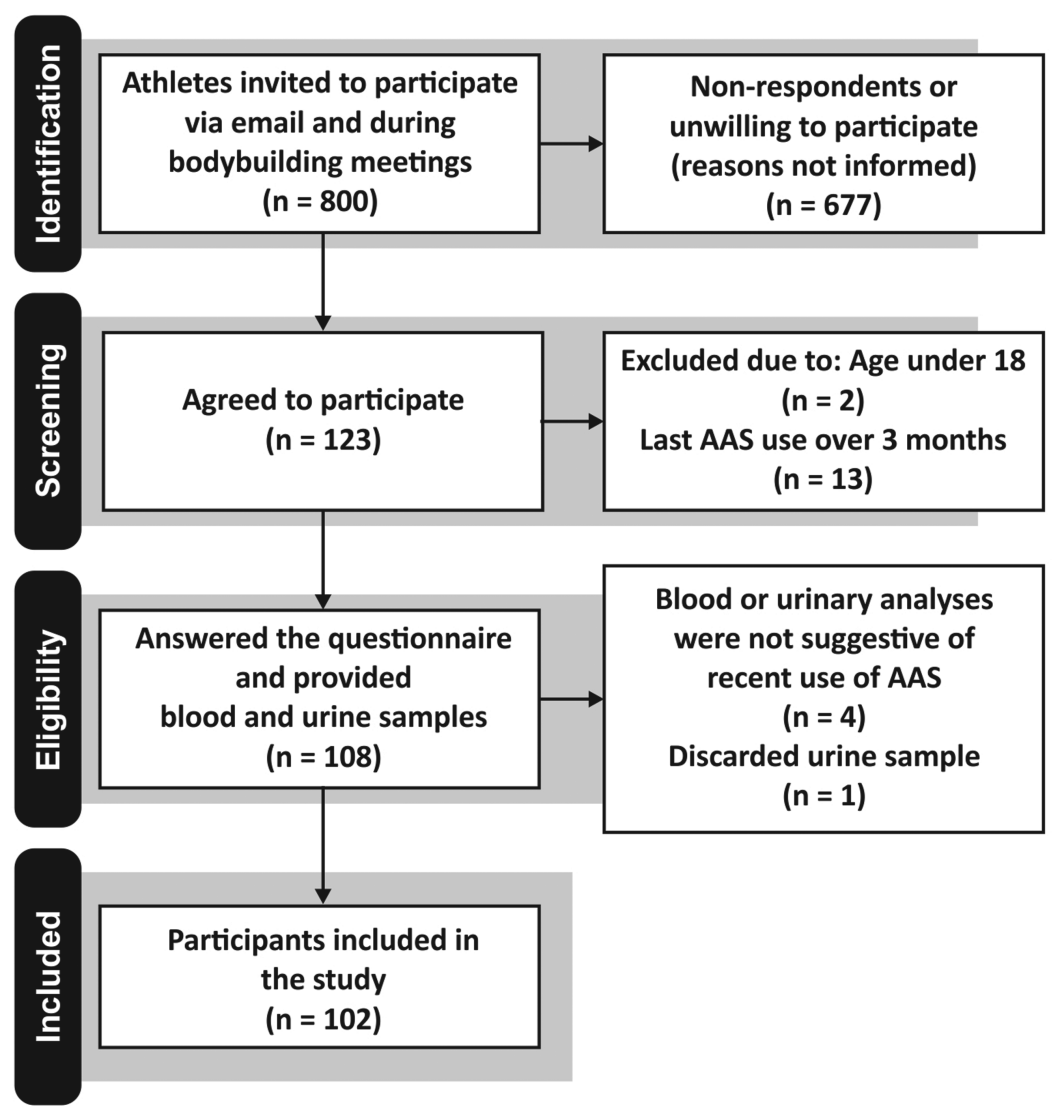
Participant's selection flowchart

A cross‐sectional study was conducted to investigate the use of AAS and psychiatric symptoms and among bodybuilders. Inclusion criteria were being 18 years old or more, consider oneself a bodybuilder, reporting the use of AAS in the last 3 months and agree with the terms of the Participant Information Sheet (PIS). Participants were recruited and took part in the study during non‐competitive meetings of Rio de Janeiro's Bodybuilding and Fitness Federation (IFBB‐RIO, Brazil) following a non‐probabilistic, purposeful sampling method. Participants did not receive financial incentives to take part in the study. Those who agreed completed a paper‐based questionnaire under supervision and provided blood and urine samples. Participants received the results of their blood exams and advice on adverse health conditions reported during the study. Urinary analysis were performed by the Brazilian Laboratory of Doping Control (LBCD), a laboratory accredited by the World Anti‐Doping Association (WADA). Results of urinary analysis were not informed to participants, in compliance with WADA regulations. Eight hundred bodybuilding athletes were invited and 123 (15.4%) agreed to take part in the study. To be included in the analysis, participants had to fulfil at least one of the following criteria suggestive of recent use of AAS: (1) Meet any criteria of WADA's suspicious steroid profile^24^ and (2) have a seric concentration of total testosterone (total‐T), follicle‐stimulating hormone (FSH) or luteinizing hormone (LH) below or above reference limits. After screening, 103 eligible participants were included in the study (Figure 1). This study was approved by the Brazilian Committee for Ethics in Research, reference CAAE 69103617.0.0000.5263.

### 2.2 Data collection

Data was collected on five different occasions between September 2017 and May 2018. To assure a sensible time for the collection of data and safe storage of urine samples, sessions of data collection were scheduled with a minimum of 10 and a maximum of 40 participants. Participants answered a questionnaire about the use of AAS and other substances in the last 3 months, psychiatric symptoms and temperament. After responding the questionnaire, male and female participants were separately directed to the exam room where a phlebotomist collected blood samples via cubital venipuncture and participants were asked to provide a sample of approximately 100 mL of urine. The first authour supervised the answering of questionnaires and collection of samples.

### 2.3 Questionnaires

Questionnaires were anonymised with a unique reference number. Participants were asked to inform their age, sex, date of birth and if they were involved in bodybuilding competitions. Participants were asked to inform when they have used AAS for the first time in their lives (AAS years), types and number of AAS (AAS‐number) used in the last 3 months, weekly dosage of AAS (AAS‐dose), use of other image and performance‐enhancing drugs (IPED) and recreational drugs. Assessment of psychiatric symptoms was based on self‐reported presence of symptoms of insomnia, aggressiveness, agitation and depression in the last 3 months, scores on Hamilton's anxiety scale (HAM‐A) and in the Buss‐Perry aggressiveness questionnaire, short form (BPQ). HAM‐A consists of 14 items aimed to assess the severity of symptoms of psychic and somatic anxiety such as anxious mood, tension, difficulties in concentration, respiratory and autonomic symptoms.^25^, ^26^ BPQ measures four traits of aggressiveness and an overall score: verbal aggressiveness, assessed with questions such as ‘I can't help getting into arguments when people disagree with me’; physical aggressiveness, assessed with questions such as ‘Given enough provocation, I may hit another person’; anger, representing an emotional component related to psychological arousal and preparation for aggression—for example, ‘I flare up quickly’, ‘I have trouble controlling my temper’; and hostility, which involves a cognitive perception of injustice and mistrust—for example, ‘I feel I have gotten a raw deal out of life’, ‘I know people are talking about me behind my back’.^27^, ^28^ In this study, scores on the HAM‐A and BPQ scale were ranked as follows. HAM‐A: none to mild, 0–14 points; moderate to high, 15 points or more. BPQ: none to mild = 1.0–2.3 points; moderate to high, 2.4 points or more. The predominant temperament of participants was assessed using the BRIEF‐TEMPS scale as being depressive (sad, pessimist), cyclothymic (restless, prone to mood swings), irritable (grumpy, easily angered), hyperthymic (upbeat, overenergetic and overconfident), anxious (prone to unreasonable fears and somatic reactions to negative emotions), worring (insecure, overly concerned) or mixed—when more than one temperament achieves the higher score.^29^ All the psychometric scales used in this study had validated versions in Portuguese.^30^, ^31^, ^32^

### 2.4 Screening for recent use of androgenic‐anabolic steroids

Blood samples were collected by a phlebotomist and immediately taken to a third‐part laboratory for analysis of seric hormones using immunoassay methods. Reference limits (RL) adopted in the screening of recent AAS use were those informed by the laboratory: Total‐T, males: 241.0 to 827.0 ng/dl; females: 14.0 to 76.0 ng/dl; FSH, males: 1.4 to 18.1 mUI/ml; females: 1.5 to 10.2 mUI/ml. LH, males: 1.5 to 9.3 mUI/ml; females: 0.5 to 16.9 mUI/mL. RL for FSH and LH in women were the lower limit of luteal phase and the upper limit of follicular phase. Urine samples were marked with participant's age and sex and sent to the LBCD for analysis of urinary androgens. RLs of concentrations and ratios of urinary (u) androgens were based on WADA's suspicious steroid profile^24^: Testosterone (u‐T) and epitestosterone (u‐Epi): greater than 200 ng/ml in males, 50 ng/ml in females; u‐T/E ratio greater than 4.0; androsterone (u‐A) and etiocholanolone (u‐Eti): greater than 10,000 ng/ml; u‐A/T ratio greater than 2.4; 5α‐androstane, 3α‐17β, diol (u‐5α) greater than 250 ng/ml in males or greater than 150 ng/ml in females, combined with a u‐5α/Epi ratio greater than 10 in either sex; u‐5α/u‐5β ratio greater than 2.4. Samples were also screened for the presence of metabolites of AAS. A complete description of methods of urinary analysis can be found in supporting information.

### 2.5 Statistical analyses

Tests of normality—Kolmogorov–Smirnov, Shapiro–Wilk and histograms—indicated that the study's variables were not normally distributed, therefore non‐parametric analyses were performed. Mann–Whitney U and Person chi‐square tests were used to compare data from male and female participants and the null hypothesis was considered rejected if *p* < 0.05. Taking into account that men and women have different profiles of endogenous androgens^33^ and their bodies respond differently to AAS,^34^ descriptive and association analysis were performed separately for each sex. Descriptive analyses describe and compare male and female participants' characteristics, self‐reported use of AAS and other substances, psychiatric symptoms and temperaments. Statistical analyses were performed in IBM‐SPSS® v27.

## 3 RESULTS

According to the parameters of WADA's steroid profile (WSP), 77 (72.0%) of members of the screening sample had suspicious use of AAS and 88 (82.2%) had concentration of seric hormones outside RL (Table 1). Among 90 (84.1%) members of the screening sample who tested positive for AAS metabolites, the majority had abnormal values in WSP (88.3%) and seric hormones (86.4%). The analysis of seric hormones was the test indicating suspicious use of AAS for a larger proportion of males (93.5%), while the detection of AAS metabolites was the test indicating suspicious use of AAS for a larger proportion of females (86.7%). The combination of these methods led to the inclusion of 95.4% of members of the screening sample.

**TABLE 1:** Screening for recent use of AAS

	Total	Males	Females
**Screening criteria, n (%)**	107	77 (72.0)	30 (28.0)
**WADA's steroid profile (WSP)**			
u‐T	34 (31.8)	27 (35.1)	7 (23.3)
u‐Epi	0 (0.0)	0 (0.0)	0 (0.0)
u‐A	6 (5.6)	6 (7.8)	0 (0.0)
u‐Eti	8 (7.5)	8 (10.4)	0 (0.0)
u‐5α^a^	18 (16.8)	17 (22.1)	1 (3.3)
u‐T/Epi	75 (70.1)	63 (81.8)	12 (40.0)
u‐5α/5β	4 (3.7)	4 (5.2)	0 (0.0)
u‐Andro/T	35 (32.7)	31 (40.3)	4 (13.3)
u‐5α/Epi^a^	90 (84.1)	64 (83.1)	26 (86.7)
u‐5α and 5α/Epi	16 (15.0)	15 (19.5)	1 (3.3)
Any WSP	77 (72.0)	65 (84.4)	12 (40.0)
**Seric hormones**			
total‐T	63 (58.9)	58 (75.3)	5 (16.7)
FSH	78 (72.9)	66 (85.7)	12 (40.0)
LH	78 (72.9)	68 (88.3)	10 (33.3)
Any seric hormones	88 (82.2)	72 (93.5)	16 (53.3)
**AAS metabolites**	90 (84.1)	64 (83.1)	26 (86.7)
Any WSP^b^	68 (88.3)	57 (89.1)	11 (42.3)
Seric hormones^b^	76 (86.4)	62 (96.9)	14 (53.8)
**Included participants**	103 (96.3)	75 (97.4)	28 (93.3)

*Note*: *n* (%): Number of participants and percentages of those with abnormal values among each group.

^a^ Separated vales of u‐5a and 5a/Epi are not included in WSP.

^b^ Participants with abnormal WSP or seric hormones among those who tested positive for AAS metabolites. u‐T: urinary testosterone. u‐Epi: urinary epitestosterone. u‐Andro: urinary androsterone. u‐Eti: urinary etiocholanolone. u‐5α: urinary 5α‐androstane, 3α‐17β, diol. u‐5β: urinary 5β‐androstane, 3α‐17β, diol. Total‐T: total seric testosterone. FSH: follicle‐stimulating hormone. LH: luteinising hormone.

Among 103 participants with suspicious use of AAS confirmed by the screening methods, 75 (72.8%) were males and 28 (27.2%) females (Table 2). Unless otherwise noted, there was no statistically significant difference between males and females' characteristics. The majority of participants (84.5%) were competitive bodybuilders, with an average age of 33 years old (minimum = 18, maximum = 58). The majority of participants used AAS for more than 1 year, and a significantly higher proportion of males (32.0%) used AAS for more than 10 years when compared to females (7.1%). On average, male participants used higher doses and a larger number of different AAS in the last 3 months. The most common AAS used by participants were testosterone esters (71.8%), stanozolol (41.7%) and trenbolone (29.1%). Higher proportions of males used testosterone esters (89.3%) and trenbolone (37.3%) when compared to females. Higher proportions of females used oxandrolone (67.9%) and methenolone (32.1%). Among other IPEDs used by participants in the last 3 months, the most common were clenbuterol (45.6%), antiestrogens (38.8%) and human growth hormone (GH; 33.0%). A higher proportion of males used antiestrogens (49.3%) and thyroid hormones (25.3%). Among the participants who reported the use of recreational in the last 3 months, the most common was cannabis (17.5%), followed by 3,4‐Methylenedioxy methamphetamine (MDMA; 7.8%) and sedatives (6.8%).

**TABLE 2:** Summary characteristics

	Total	Males	Females	
**Characteristics, *n* (%)**	103	75 (72.8)	28 (27.2)	**Comparisons**
**Competitive bodybuilders**	87 (84.5)	64 (85.3)	23 (82.1)	*χ* ^2^ = 0.158, df = 2, *p* = 0.691
**Age: *median (min–max)***	*33 (18–58)*	*32 (18–58)*	*33.5 (20–44)*	U = 1098.5, p = 0.719
18–25	19 (18.4)	16 (21.3)	3 (10.7)	*χ* ^2^ = 1.878, df = 2, *p* = 0.391
26–33	33 (32.0)	22 (29.3)	11 (39.3)	
34–58	51 (49.5)	37 (49.3)	14 (50.0)	
**AAS‐years** ^a^				*χ* ^2^ = 7.604, df = 2, *p* = 0.022*
less than 12 months	26 (25.2)	19 (25.3)	7(25.0)	
1 to 10 years	51 (49.5)	32 (42.7)	19 (67.9)	
more than 10 years	26 (25.2)	24 (32.0)	2 (7.1)	
**AAS‐dose (mg/week)** ^b^				*χ* ^2^ = 12.991, df = 3, *p* = 0.005**
up to 410	25 (24.3)	12(16.0)	13 (46.4)	
420–875	26 (25.2)	20 (26.7)	6 (21.4)	
885–1510	26 (25.2)	19 (25.3)	7 (25.0)	
1555–3600	26 (25.2)	24 (32.0)	2 (7.1)	
**AAS‐number** ^c^				*χ* ^2^ = 12.365, df = 2, *p* = 0.002**
1–2	33 (32.0)	17 (22.7)	16 (57.1)	
3–5	47 (45.6)	37 (49.3)	10 (35.7)	
6 or more	23 (22.3)	21 (28.0)	2 (7.1)	
**Type of AAS**				
**testosterone (esters, Sustanon®)**	74 (71.8)	67 (89.3)	7 (25.0)	*χ* ^2^ = 41.716, df = 1, *p* < 0.001**
**stanozolol (Winstrol®)**	43 (41.7)	29 (38.7)	14 (50.0)	*χ* ^2^ = 1.077, df = 1, *p* = 0.299
**trenbolone**	30 (29.1)	28 (37.3)	2 (7.1)	*χ* ^2^ = 9.002, df = 1, *p* = 0.003**
**drostanolone (Masteron®)**	30 (29.1)	25 (33.3)	5 (17.9)	*χ* ^2^ = 2.366, df = 1, *p* = 0.124
**oxandrolone (Anavar®)**	29 (28.2)	10 (13.3)	19 (67.9)	*χ* ^2^ = 29.964, df = 1, *p* < 0.001**
**boldenone (Equipoise®)**	21 (20.4)	16 (21.3)	5 (17.9)	*χ* ^2^ = 0.152, df = 1, *p* = 0.697
**nandrolone (Deca‐Durabolin®)**	16 (15.5)	10 (13.3)	6 (21.4)	*χ* ^2^ = 1.018, df = 1, *p* = 0.313
**methandrostenolone (Dianabol®)**	10 (9.7)	9 (12.0)	1 (3.6)	*χ* ^2^ = 1.652, df = 1, *p* = 0.199
**methenolone (Primobolan®)**	13 (12.6)	4 (5.3)		*χ* ^2^ = 13.288, df = 1, *p* < 0.001**
**oxymetholone (Anadrol®)**	4 (3.9)	3 (4.0)	1 (3.6)	*χ* ^2^ = 0.010, df = 1, *p* = 0.920
**mesterolone (Pro‐Viron®)**	2 (1.9)	2 (2.7)	0 (0.0)	*χ* ^2^ = 0.761, df = 1, *p* = 0.383
**bio‐identic testosterone**	2 (1.9)	2 (2.7)	0 (0.0)	*χ* ^2^ = 0.761, df = 1, *p* = 0.383
**Other IPEDs**				
**Clenbuterol**	47 (45.6)	35 (46.7)	12 (42.9)	*χ* ^2^ = 0.119, df = 1, *p* = 0.730
**Antiestrogens**	40 (38.8)	37 (49.3)	3 (10.7)**	*χ* ^2^ = 12.801, df = 1, *p* < 0.001**
**Human growth hormone**	34 (33.0)	26 (34.7)	8 (28.6)	*χ* ^2^ = 0.343, df = 1, *p* = 0.558
**Thyroid hormones**	21 (20.4)	19 (25.3)	2 (7.1)*	*χ* ^2^ = 4.156, df = 1, *p* = 0.041*
**Ephedrine**	12 (11.7)	10 (13.3)	2 (7.1)	*χ* ^2^ = 0.759, df = 1, *p* = 0.384
**Human chorionic gonadotropin**	3 (2.9)	3 (4.0)	0 (0.0)	*χ* ^2^ = 1.154, df = 1, *p* = 0.283
**Any other IPED**	81 (78.6)	62 (82.7)	19 (67.9)	*χ* ^2^ = 2.662, df = 1, *p* = 0.103
**Recreational drugs**				
**Cannabis**	18 (17.5)	13 (17.3)	5 (17.9)	*χ* ^2^ = 0.004, df = 1, *p* = 0.950
**MDMA**	8 (7.8)	6 (8.0)	2 (7.1)	*χ* ^2^ = 0.021, df = 1, *p* = 0.885
**Sedatives**	7 (6.8)	4 (5.3)	3 (10.7)	*χ* ^2^ = 0.932, df = 1, *p* = 0.334
**LSD**	4 (3.9)	3 (4.0)	1 (3.6)	*χ* ^2^ = 0.010, df = 1, *p* = 0.920
**Inhalants**	2 (1.9)	2 (2.7)	0 (0.0)	*χ* ^2^ = 0.761, df = 1, *p* = 0.383
**Cocaine**	1 (1.0)	1 (1.3)	0 (0.0)	*χ* ^2^ = 0.377, df = 1, *p* = 0.539
**Any recreational drug**	25 (24.3)	17 (24.0)	7 (25.0)	*χ* ^2^ = 0.011, df = 1, *p* = 0.916

*Note*: *n* (%): Number of participants and percentages among each group. *χ* ^2^: Pearson's chi‐square. df: degrees of freedom. U: Mann‐Whitey U. IPED: Image and performance‐enhancement drugs. MDMA: 3,4‐Methylenedioxymethamphetamine. LSD: Lysergic acid diethylamide.

^a^ Time since the first use of AAS, in years.

^b^ Estimated weekly dose of AAS in the last 3 months in mg/week.

^c^ Different number of AAS used in the last 3 months.

* *p* < 0.05.

** *p* < 0.01.

### 3.1 Psychiatric symptoms and temperaments

As seen in Table 3, the majority of participants (61.2%) presented some of the investigated psychiatric symptoms in the last 3 months. The most common symptoms were agitation (35.9%) and insomnia (35.0%), presented by about one‐third of participants. About one‐third of participants (31.1%) presented moderate to high scores of anxiety in the HAM‐A scale. Although the difference between sexes was not considered statistically significant, a higher proportion of females (42.9%) presented moderate to high scores of anxiety when compared to males (26.7%). Only a minority of participants presented moderate to high scores of aggressiveness in the BPQ scale. Among the traits of aggressiveness, a slightly higher proportion of participants had moderate to high scores on the anger trait (15.5%). The majority of participants (73.8%) presented a predominance of the hyperthymic temperament. Since there was no assessment of temperament before the participants started using AAS, it is not possible to affirm that the use of AAS had an influence in their temperament, although it befits the description of some behavioural effects of AAS such as increased energy and confidence.^22^, ^35^

**TABLE 3:** Psychiatric symptoms and temperaments

	Total	Males	Females	
**Symptoms and temperaments: n (%)**	103	75 (72.8)	28 (27.2)	**Comparisons**
Agitation	37 (35.9)	27 (36.0)	10 (35.7)	*χ* ^2^ = 0.001, df = 2, *p* = 0.979
Insomnia	36 (35.0)	25 (33.3)	11 (39.3)	*χ* ^2^ = 0.318, df = 1, *p* = 0.573
Increased aggressiveness	25 (24.3)	20 (26.7)	5 (17.9)	*χ* ^2^ = 0.861, df = 1, *p* = 0.354
Depression	11 (10.7)	8 (10.7)	3 (10.7)	*χ* ^2^ = 0.000, df = 1, *p* = 0.994
Any of the above	63 (61.2)	47 (62.7)	16 (57.1)	*χ* ^2^ = 0.262, df = 1, *p* = 0.609
**HAM‐A: moderate to severe**	32 (31.1)	20 (26.7)	12 (42.9)	*χ* ^2^ = 2.496, df = 2, *p* = 0.114
**BPQ: moderate to high**				
Verbal aggression	8 (7.8)	6 (8.0)	2 (7.1)	*χ* ^2^ = 0.021, df = 1, *p* = 0.885
Physical aggression	8 (7.8)	7 (9.3)	1 (3.6)	*χ* ^2^ = 0.945, df = 1, *p* = 0.331
Anger	16 (15.5)	12 (16.0)	4 (14.3)	*χ* ^2^ = 0.046, df = 1, *p* = 0.831
Hostility	9 (8.7)	6 (8.0)	3 (10.7)	*χ* ^2^ = 0.188, df = 1, *p* = 0.664
BPQ − total	13 (12.6)	9 (12.0)	4 (14.3)	*χ* ^2^ = 0.097, df = 1, *p* = 0.756
**BRIEF‐TEMPS**				
Hyperthymic	79 (73.8)	57 (76.0)	19 (67.9)	*χ* ^2^ = 0.699, df = 1, *p* = 0.403
Worring	11 (10.7)	9 (12.0)	2 (7.1)	*χ* ^2^ = 0.504, df = 1, *p* = 0.478
Cyclothhymic	5 (4.9)	3 (4.0)	2 (7.1)	*χ* ^2^ = 0.436, df = 1, *p* = 0.509
Anxious	0 (0.0)	0 (0.0)	0 (0.0)	n/a
Irritable	0 (0.0)	0 (0.0)	0 (0.0)	n/a
MIxed	11 (10.7)	6 (8.0)	5 (17.9)	*χ* ^2^ = 2.077, df = 1, *p* = 0.150

*Note*: *n* (%): Number of participants and percentages among each group. *χ* ^2^: Pearson's chi‐square. df: degrees of freedom.

* *p* < 0.05.

** *p* < 0.01.

As seen in Table 4, there was no association between AAS‐doses and the presence of psychiatric symptoms or scores on the scales HAM‐A and BPQ. Some associations were found between scores on the BPQ scale and AAS‐years. Among the small number of participants with moderate to high scores on verbal aggression, anger and total score, none had used AAS for more than 10 years.

**TABLE 4:** Association analyses

	AAS‐dose (mg/week)			AAS‐years			
	21–880	900–3,600		< 12 months	1–10 years	> 10 years	
**Symptoms: n (%)**	53 (51.5)	48 (48.5)	**Comparisons**	26 (25.2)	51 (49.5)	26 (25.2)	**Comparisons**
Agitation	17 (32.1)	20 (40.0)	*χ* ^2^ = 0.702, df = 2, *p* = 0.402	9 (34.6)	16 (31.4)	12 (46.2)	*χ* ^2^ = 1.660, df = 2, *p* = 0.436
Insomnia	18 (34.0)	18 (36.0)	*χ* ^2^ = 0.047, df = 1, *p* = 0.828	8 (30.8)	20 (39.2)	8 (30.8)	*χ* ^2^ = 0.808, df = 2, *p* = 0.668
Increased aggressiveness	10 (18.9)	15 (30.0)	*χ* ^2^ = 1.735, df = 1, *p* = 0.188	5 (19.2)	15 (29.4)	5 (19.2)	*χ* ^2^ = 1.735, df = 2, *p* = 0.188
Depression	5 (9.4)	6 (12.0)	*χ* ^2^ = 0.178, df = 1, *p* = 0.673	4 (15.4)	4 (7.8)	3 (11.5)	*χ* ^2^ = 1.054, df = 2, *p* = 0.590
Any of the above	32 (60.4)	31 (62.0)	*χ* ^2^ = 0.029, df = 1, *p* = 0.866	15 (57.7)	31 (60.8)	17 (65.4)	*χ* ^2^ = 0.330, df = 2, *p* = 0.848
**HAM‐A: moderate to severe**	15 (28.3)	17 (34.0)	*χ* ^2^ = 0.390, df = 2, *p* = 0.532	8 (30.8)	17 (33.3)	7 (26.9)	*χ* ^2^ = 0.332, df = 2, *p* = 0.847
**BPQ: moderate to high**							
Verbal aggression	2 (3.8)	6 (12.0)	*χ* ^2^ = 2.430, df = 1, *p* = 0.119	0 (0.0)	8 (15.7)	0 (0.0)	*χ* ^2^ = 8.844, df = 2, *p* = 0.012*
Physical aggression	4 (7.5)	4 (8.0)	*χ* ^2^ = 0.007, df = 1, *p* = 0.932	2 (7.7)	5 (9.8)	1 (3.8)	*χ* ^2^ = 0.854, df = 2, *p* = 0.653
Anger	5 (9.4)	11 (22.0)	*χ* ^2^ = 3.096, df = 1, *p* = 0.078	6 (23.1)	10 (19.6)	0 (0.0)	*χ* ^2^ = 6.554, df = 2, *p* = 0.038*
Hostility	4 (7.5)	5 (10.0)	*χ* ^2^ = 0.194, df = 1, *p* = 0.660	2 (7.7)	4 (7.8)	3 (11.5)	*χ* ^2^ = 0.343, df = 2, *p* = 0.843
BPQ − total	6 (11.3)	7 (14.0)	*χ* ^2^ = 0.167, df = 1, *p* = 0.682	3 (11.5)	10 (19.6)	0 (0.0)	*χ* ^2^ = 6.040, df = 2, *p* = 0.049*

*Note*: *n* (%): Number of participants and percentages among each group. *χ* ^2^: Pearson's chi‐square. df: degrees of freedom.

* *p* < 0.05.

** *p* < 0.01.

## 4 DISCUSSION

To the best of our knowledge, this is the first study to use WADA's steroid profile of subjects suspicious of AAS abuse combined with the detection of AAS metabolites and seric hormones to screen for AAS use in a cohort of male and female bodybuilders. Differences between the numbers of males and females with abnormal values in each of the parameters highlights the importance of effective and accessible methods of doping detection.^36^ We believe that the refinement of these methods can contribute to anti‐doping screening in recreational sport settings and to the quality of ecological studies with people using AAS. The majority of participants of this study were competing bodybuilders and experienced AAS users, suggesting a reasonable experience with the management of adverse effects. The higher number of long‐term male AAS users in this sample is in conformity with a higher prevalence of AAS use among men,^37^ whilst the significant proportion of female AAS users corroborates previous findings reporting an increasing prevalence of AAS use among women.^38^, ^39^ This tendency is corroborated by our findings of similar proportions of ‘new users’—those using AAS for less than 1 year—and the much higher proportion of males who used AAS for more than 10 years. The majority of male participants reported AAS‐doses five‐to‐eight times higher than females, similarly as described in previous studies.^38^, ^40^, ^41^ Some of the AAS used by most male and female participants were also remarkably different. The AAS used by the majority of males, Sustanon® (a combination of four testosterone esters), is an injectable AAS mixture available since 1970, with considerable anabolic and androgenic properties.^42^ Trenbolone, also used by a higher proportion of males, is considered a potent AAS, with a high affinity to the androgen receptor^43^ and anecdotally associated to an increased risk of clinical and behavioural adverse effects.^44^ As seen in this study, Anavar® is an AAS commonly used by women^34^, ^38^, ^41^—which seems to be related to its oral use and alleged lower androgenic potential. Polypharmacy has been described as a common practice among people using AAS, and our findings corroborate the concept that the concurrent of AAs and other substances must be understood in a functional context.^45^, ^46^, ^47^, ^48^ This could explain, in the one hand, a greater prevalence of use of IPEDs such as Clenbuterol and thyroid hormones—used to enhance focus and motivation during strength training, increase metabolism rates and promote weight loss^48^ and GH, which have mild anabolic properties but contributes to weight loss and the visualization of muscle groups in bodybuilding competitions.^49^, ^50^ In the other hand, only a minority of participants reported the use of recreational drugs. The use of recreational drugs was reported by a minority of participants, which could be associated with the avoidance of drugs adopted by some competitive bodybuilders.^23^ However, substances such as cannabis and sedatives have been reported as been used in an attempt to reduce adverse effects of AAS such as insomnia and anxiety.^48^, ^51^ The similar proportions of male and female participants of this study who reported the use of recreational drugs also corroborate a similarity of practices and lifestyle among.^21^ The majority of male and female participants presented symptoms of either insomnia, aggressiveness, agitation or depression in the last 3 months, as seen in other studies with AAS users.^4^, ^5^, ^7^, ^14^ Likewise, about one‐third of participants presented scores on HAM‐A equivalent to moderate to severe symptoms of anxiety. These findings highlight the importance of addressing the mental health of people using AAS, as it has been considered one of the main factors leading to satisfaction with medical support by that population.^52^ Besides, as people using AAS become aware of these risks and feel comfortable seeking support for mental health conditions with knowledgeable health professionals, the greater are the chances that more severe outcomes can be prevented. Although about one‐quarter of participants had reported increased aggressiveness in the last 3 months, only a minority of them scored moderate or high in the BPQ‐SF scale. A possible explanation for this discrepancy would be that the aggressiveness experienced by some participants was perceived as a transitory condition that was no longer present when the questionnaires were filled.

This study observed a high prevalence of psychiatric effects among AAS users, which were not associated with the doses of AAS taken by the participants and negatively associated with their time of AAS use. Instead of contradicting the evidence of a dose‐related association between the use of AAS and aggression seen in randomly controlled trials – as summarized by Chegeni et al.^8^—we believe that these results highlight the complexity of ecological observations of AAS use. A large body of literature indicates that factors other than the sheer exposure to AAS can influence the onset and severity of adverse effects, such as individual vulnerabilities, distinct effects of different AAS and strategies adopted by AAS users to treat and prevent adverse effects.^5^, ^53^, ^54^, ^55^, ^56^, ^57^ Besides, symptoms such as insomnia and anxiety among this population could be associated with training routines and the use of other substances that were not investigated by this study. As previously described, bodybuilders frequently share strategies and lifestyles perceived as necessary to cope with the huge number of physical, mental and chemical stressors sometimes associated with this discipline.^23^, ^44^, ^58^, ^59^, ^60^ As the majority of participants of this study were experienced AAS users and bodybuilding athletes, it is possible that those able to endure higher doses and longer periods of AAS use are those who are less prone to experience—or more able to manage—adverse effects of AAS, highlighting the risk of a selection bias. Other limitations of this study include the small number of athletes selected to participate and the absence of a control group of non‐AAS‐using bodybuilding athletes. Analysis of seric hormones were performed using immunoassay methods, less accurate then the gold standard of liquid chromatography coupled to mass spectrometry.^61^ Psychiatric symptoms in this study were only superficially evaluated, with no diagnostic value or measurements of intensity, frequency or details about the presentation of these conditions.

## 5 CONCLUSION

This study support findings of a high prevalence of psychiatric symptoms among bodybuilding athletes using AAS and a growing prevalence of AAS use among women. Screening methods must be refined by further analysis to improve the detection of recent use of AAS in the general population, namely in clinical and recreational sport settings. The lack of an association between psychiatric symptoms and doses of AAS suggest the presence of other factors influencing the ecological observation of these effects among people AAS. Further studies are necessary to understand the impact of factors such as individual vulnerabilities, effects of different AAS and harm‐reduction strategies on the occurrence of psychiatric effects among people using AAS. Our findings highlight the importance of mental health awareness among people using AAS and health professionals supporting that population.

## Supporting information


**Data S1.** Supporting Information

## Data Availability

The data that supports the findings of this study are available in the supplementary material of this article.
