# Supplementary material for: No association between psychiatric symptoms and doses of anabolic steroids in a cohort of male and female bodybuilders
Source: Drug Test Anal. 2022 Feb 21;14(6):1079–88. doi: 10.1002/dta.3230 (PMC9303351; doi:10.1002/dta.3230)
Supplement: Supplementary file 1 — Data S1. Supporting Information [file DTA-14-1079-s001.pdf]

Psychiatric symptoms, seric and urinary measurements of anabolic steroid use in a cohort of male and female bodybuilders

Supplementary file 1: Methods of urinary analysis

Urine samples were stored under 4°C until delivery to the BLDC, no more than 48h after collection. Urine sample preparation was based on enzymatic hydrolysis and liquid-liquid extraction of AAS. Aliquots of 2 mL urine samples were spiked with 40 µL of internal standard (Table 1).

Table 1: Internal standard composition

Deuterated steroid	Concentration (ng/µL)
**Epitestosterone-D3**	0.75
**Testosterone-D3**	3.00
**Etiocholanolone -D4**	25.00
**Androsterone glucuronide-D5**	25.00
**5α-Androstanediol-D5**	4.00
**5β-Androstanediol-D4**	9.00

During the initial testing procedure (screening), the steroid profile was estimated by single-point calibration. Calibration samples, ie, quality control of endogenous steroids (CQENDO) were prepared by spiking synthetic urine with endogenous steroids standards in their free form. Nominal final concentrations of the compounds for the CQENDO samples are presented in Table 2.

Table 2: CQENDO composition

Endogenous steroids	Concentration (ng/µL)
11-Cetoetiocholanolone 1000	1000
16-Androstenol 500	500
Tetrahydrocortisol 1000	1000

Pregnanediol 1000	1000
Prasterone 400	400
Dihydrotestosterone 400	400
11βOH-Etiocholanolone 200	200
5α-Androstanediol 180	180
5β-Androstanediol 80	80
Epitestosterone 40	40
Testosterone 40	40
Etiocholanolone 2000	2000
Androsterone	2000

The pH was adjusted with 750 µL of 0.8 M phosphate buffer, and 50 µL of β-glucuronidase obtained from *Escherichia coli* (*E.coli*) were added. Incubated at 50°C for 1 hour, and then added 500 µL of aqueous buffer solution containing K_2CO_3 / $KHCO_3$ 20% (w/w) and 4 mL of methyl tert-butyl ether (TBME). The mixture was stirred for 5 min and centrifuged at 3000 rpm for 5 min. After phase separation, the organic phase was evaporated to dryness under nitrogen and dried in a vacuum oven for 30 min at room temperature. Finally, the residue was derivatized with 100 µL of N-methyl-N-(trimethylsilyl) trifluoroacetamide (MSTFA)–NH_4I–2-mercaptoethanol (1000:2:6, v/w/v) 60°C for 20 minutes. Aliquots of 2 µL were injected in splitless mode into the triple quadrupole system (GC-QqQ) operated in multiple reaction monitoring modes. The analysis was performed using a Gas Chromatograph (GC) Trace 1310 (Thermo Scientific) and interfaced with a mass spectrometer TSQ 8000 (Thermo Scientific), column 100% methylpolysiloxane phase (17m×0.20mm×0.11 µm).

List of the reagents: Methanol (pesticide grade), ethyl acetate (GC grade), acetone (GC grade), and tert butyl methyl ether (GC grade) were purchased from Tedia (Fairfield, OH, USA). N-methyl-N-(trimethylsilyl)-trifluoroacetamide, 2-mercaptoethanol and ammonium iodide were purchased from Sigma Aldrich (St Louis, MO, USA). Sodium dihydrogen phosphate monohydrate, di-sodium hydrogen phosphate, potassium

carbonate, and potassium hydrogen carbonate were purchased from Merck KGaA (Darmstadt, Germany). Beta glucuronidase from *E. coli* was purchased from Roche Diagnostics GmbH (Mannheim, Germany). Compressed helium (99.999% purity) and argonium (99.999% purity) gases were purchased from White Martins (São Paulo, Brazil). All reference materials were purchased from the National Measurement Institute (Australia).

GC electron impact source mass spectrometry (GC-EI-MS/MS) conditions: The oven temperature program was 140 to 230°C at 40°C/min, 230 to 280°C at 3°C/min, 280 to 300°C and held at 300°C for 3 min. The transfer line was set to 300°C and the ion source was set to 320°C. Electron ionization was performed using electron energy of 70eV.

The collision energies were optimized according to the software Auto SRM. The dwell time was set to reach ten points across the peak for the narrowest peak.

Liquid chromatography (LC) and high-resolution mass spectrometry (LC-HRMS): LC separations were performed on Dionex UltiMate 3000 UHPLC system (ThermoScientific, using a Syncronis C 18 column (50 x 2.1 mm x 1.7 µm) maintained at 40 ºC. The mobile phase A (water, 0,1% formic acid and 5 mM ammonium formate) and mobile phase B (methanol and 0,1% formic acid) was employed in the following gradient of B mobile phase: 0 min, 5%; 0,3 min, 5%, 0,5 min, 10%; 1 min 25%; 6 min, 90%; 8 min, 100%; 9 min, 100%; 9,1 min, 5%; 11 min, 5%. The flow rate was 400 µL/min and the injection volume was 10 µL. For high-resolution mass spectrometry analysis was used a QExactiveTM Plus Orbitrap mass spectrometer (MS) (ThermoFisher Scientific, Bremen, Germany) in positive mode ionization. The mass detection range was m/z 100-900 in data-dependent acquisition mode for MS^2 fragmentation experiments. The automatic gain control (AGC) target was set to 1e6 and maximum ion time (IT) was set to 100 ms. Sheath gas flow rate and auxiliary gas flow rate were set to 60 and 20 respectively, spray voltage 3.90 kV, capillary temperature was 380 ºC , S-lens radio frequency (RF) level 80, auxiliary gas heater temperature was 380° C.

The data were evaluated using Thermo Fisher Scientific TraceFinderTM 3.2.512.0 software (Thermo Fisher Scientific, Waltham, MA, USA).
